# Orlistat Confers Neuroprotection in Traumatic Brain Injury by Modulating Microglial Lipid Metabolism

**DOI:** 10.3390/cells14181469

**Published:** 2025-09-19

**Authors:** Chenxuan Yu, Yu Ni, Yuxuan Xiong, Huayu Kang, Zhengqiao Jiang, Yuan Liu, Xincheng Zhang, Yanchao Liu, Kai Zhao, Sheng Wang, Chao Gan, Huaqiu Zhang

**Affiliations:** 1 Department of Neurosurgery, Tongji Hospital of Tongji Medical College of Huazhong University of Science and Technology, Wuhan 430030, China; 2 Hubei Key Laboratory of Neural Injury and Functional Reconstruction, Huazhong University of Science and Technology, Wuhan 430030, China

**Keywords:** TBI, microglial, orlistat, LPL, neuroinflammation

## Abstract

Traumatic brain injury (TBI) represents a major cause of mortality and disability worldwide, particularly affecting young adults and elderly populations. This study investigates the neuroprotective potential of orlistat (ORL), a gastrointestinal lipase inhibitor, in a murine TBI model. Behavioral, histological, and molecular analyses demonstrated that ORL significantly attenuated TBI-induced neurological damage. Microglial depletion experiments revealed that ORL’s neuroprotective effects were largely mediated through microglial modulation. In vitro and in vivo studies showed that ORL suppressed microglial activation, phagocytosis, and migration. Single-cell RNA sequencing identified upregulation of lipoprotein lipase (LPL) in a TBI-induced microglial subpopulation. Molecular docking predicted ORL-LPL binding, suggesting direct enzymatic inhibition. Transcriptomic and metabolomic analyses further revealed ORL’s modulation of microglial metabolic pathways and inflammatory responses. Our findings position ORL as a promising repurposed therapeutic for TBI through its novel mechanism of targeting microglial LPL-mediated neuroinflammation.

## 1. Introduction

Traumatic brain injury (TBI), resulting from external mechanical forces, constitutes a prevalent central nervous system disorder characterized by sudden structural damage and functional impairment [1,2]. TBI is a highly prevalent disabling and fatal disease worldwide, with an annual incidence rate of 200 to 300 cases per 100,000 people. Young and middle-aged people are prone to it due to traffic accidents and violence, while the elderly are often affected by it through falls. The risk for men is 2 to 3 times that for women. The mortality rate for severe patients is 30% to 50%, and half of the survivors have permanent neurological dysfunction [3]. The neurological damage in TBI arises through two mechanisms: primary injury from direct mechanical force and secondary injury involving oxidative stress, metabolic dysfunction, blood–brain barrier disruption, and cerebrovascular dysregulation [4]. Despite extensive research, clinically effective neuroprotective agents remain elusive.

Orlistat, an FDA-approved oral gastrointestinal lipase inhibitor, reduces dietary fat absorption by inhibiting gastric and pancreatic lipases [5]. Emerging evidence suggests that microglia—CNS-resident immune cells—undergo metabolic reprogramming toward a pro-inflammatory M1 phenotype post-TBI [6]. After TBI, lipid metabolism disorders in microglia, such as cholesterol accumulation and enhanced lipid peroxidation, aggravate damage by activating inflammatory pathways and intensifying oxidative stress. Meanwhile, the impaired lipid phagocytic function affects tissue repair and is an important regulatory target for secondary injury and prognosis [7]. We hypothesized that ORL might modulate microglial lipid metabolism to mitigate neuroinflammation. In this study, we administered ORL via intraperitoneal injection to bypass gastrointestinal absorption, demonstrating its efficacy in suppressing microglia-mediated inflammation and preserving neurological function in TBI mice. Through integrated single-cell sequencing, transcriptomics, and metabolomics approaches, we elucidated the underlying mechanisms of ORL’s central neuroprotective effects.

## 2. Results

### 2.1. Orlistat Attenuates Neurological Impairments Following TBI in Mice

**Figure 1:**
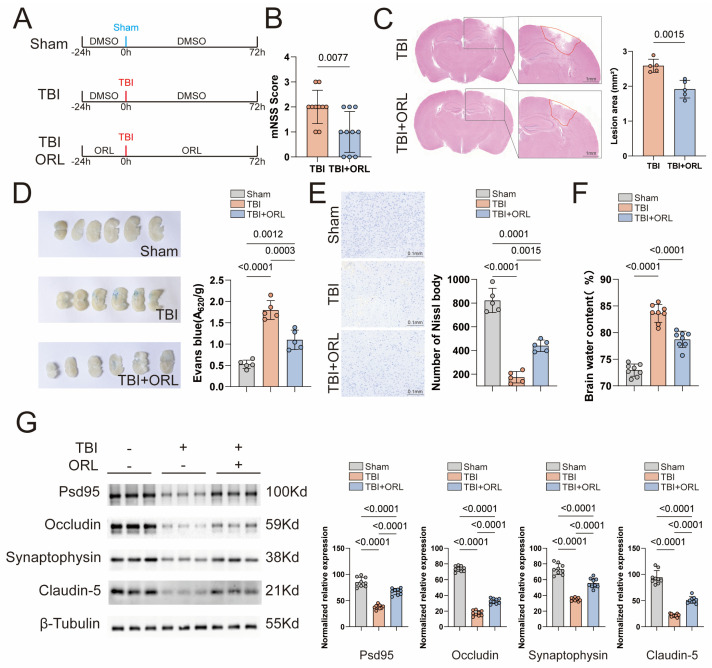
(**A**) Mice were intraperitoneally injected with DMSO or ORL 24 h before modeling and intraperitoneally injected with DMSO or ORL every day after modeling. They were sacrificed after 72 h, and brain tissues were taken for detection in subsequent experiments. (**B**) The mNSS score of TBI mice after ORL treatment, *n* =10. (**C**) The area of brain injury foci in TBI mice after ORL treatment, scale bar = 1 mm, *n* = 5. (**D**) Evans Blue was injected into TBI mice after ORL treatment to evaluate the integrity of the blood–brain barrier; on the left is the general view, and on the right are the quantified statistical results, *n* = 5. (**E**) Nissl body staining was performed on the representative brain tissues of TBI mice after ORL treatment to evaluate the survival degree of neurons at the injury site; on the left is the general view under a microscope, and on the right are the quantified statistical results, scale bar = 0.1 mm, *n* = 5. (**F**) The water content of brain tissue in TBI mice after ORL treatment, *n* = 8. (**G**) Representative Western Blot images of Psd95, Occludin5, Synaptophysin and Caludin-5 in TBI mice after ORL treatment, *n* = 9.

To explore the potential role of orlistat in TBI, we modeled TBI in mice using standard craniocerebral injury percussion as described before [8]. Meanwhile, to prevent gastrointestinal consumption of orlistat, we intraperitoneally injected orlistat into the mice every day for three days before modeling and continued intraperitoneal injection after modeling. Three days later, the brain was taken for subsequent tests (Figure 1A). 

To determine the appropriate administration concentration, we started from an initial concentration of 10 mg/kg and gradually increased it to 100 mg/kg [9]. By detecting the concentrations of IL-1β, IL-6 and TNF-α in the brains of mice, we found that when the drug concentration was increased from 10 mg/kg to 50 mg/kg, ORL could further inhibit the levels of inflammatory factors in the brains of mice after TBI (Figure S1A–C), and the mNSS score also improved significantly (Figure S1D). However, when the drug concentration was further increased to 100 mg/kg, the inhibitory effect of ORL on inflammation did not significantly improve (Figure S1A–C), and the mNSS score did not further decrease (Figure S1D). Regardless of which concentration was used, no obvious adverse reactions occurred in the mice during the administration period. Based on the above results, we ultimately chose to use a concentration of 50 mg/kg. Compared with TBI mice, ORL mice had a lower mNSS score (Figure 1B) and a lower water content in the brain (Figure 1F), indicating a significant improvement in the degree of brain tissue edema after injury. By performing HE staining on mouse brain sections, we found that the lesion defect and the area of the edema zone around the lesion in ORL mice were significantly smaller (Figure 1C). To explore the integrity of the blood–brain barrier (BBB), Evans Blue, a dye that can only enter the brain after damage to the blood–brain barrier, was injected into the tail vein of mice [10]. The results were consistent with previous ones. The content of Evans Blue in the brains of ORL mice was lower and mainly concentrated around the injury site (Figure 1D).

Neurons are the main body responsible for the executive functions of the central nervous system. Their quantity and morphology often directly reflect the severity of the injury and the difficulty of recovery in the later stage [11]. First, we used Nissl staining to detect Nissl bodies in the mouse brain, which are alkaline granules concentrated in the cell bodies or dendrites of neurons. Compared with TBI mice, ORL mice had a greater number of Nissl bodies around the injury foci, indicating that there were more functional neurons alive (Figure 1E). Then, we performed immunofluorescence staining on the brain sections. The results showed that the number of neurons in the cortex of TBI mice was significantly reduced, and a large number of axons were broken. Similarly, the number of neurons in ORL mice was greater, and although the myelin sheath was broken, it was relatively more intact (Figure S1E).

To further reflect the degree of brain tissue damage, we examined four proteins that constitute intercellular connections and play a major role in the formation of the blood–brain barrier: Psd95, Occludin, Synaptophysin and Claudin-5 [12]. Proteins were extracted from the brain tissue at the injury site after TBI for WB detection. The results showed that the contents of all four proteins decreased significantly after TBI, while they all rebounded to varying degrees in ORL mice (Figure 1G).

**Figure 2:**
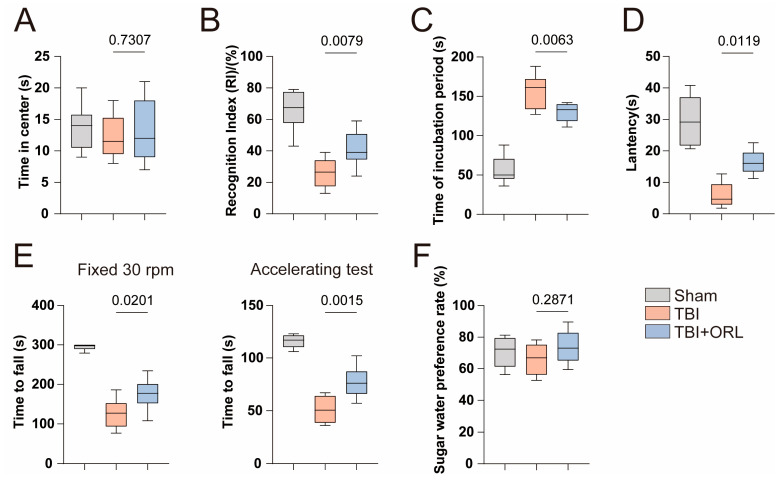
(**A**) Statistical chart of the results of the open-field test, *n* = 10. (**B**) Statistical chart of the results of the new object recognition test, *n* = 10. (**C**) Statistical chart of the results of the Barnes maze test, *n* = 10. (**D**) Statistical chart of the results of the Morris water maze, *n* = 10. (**E**) Statistical chart of the results of the rotarod test conducted on TBI mice after ORL treatment; the left chart is the result of the fixed-speed test at 30 rpm, and the right chart is the result of the acceleration test, *n* = 10. (**F**) Statistical chart of the results of the sucrose preference test conducted on TBI mice after ORL treatment, *n* = 10.

Then, we evaluated the protective effect of ORL on neurological function through behavioral experiments. For short-term memory, we conducted a new object recognition experiment three days after TBI. For long-term memory, we conducted a six-day practice on mice after TBI and carried out Barnes’ Maze and water maze experiments on the seventh day for testing. The results show that the recognition frequency of new objects in ORL mice is higher than that in TBI mice (Figure 2B and Figure S2B). Meanwhile, ORL mice can distinguish target holes more quickly in Barnes’ maze (Figure 2C and Figure S2C), and can also better remember the exits that previously existed in the third quadrant in water maze (Figure 2D and Figure S2D). It indicates that ORL has a certain degree of improvement on both short-term and long-term memory functions after TBI.

For the assessment of anxiety and depression, we conducted an open-field test and a sucrose preference test three days after TBI. Compared with TBI mice, ORL mice did not show more tendency to stay at the edge in the box (Figure 2A and Figure S2A), and they also did not consume more sucrose water (Figure 2F). It seems that the improvement effect of ORL on anxiety and depression is limited. However, it is worth noting that the impact of TBI itself on anxiety and depression is limited, and there is no significant difference in the above results between TBI mice and Sham mice.

For the motor function of mice, we conducted a rotation experiment three days after TBI, including uniform rotation and uniform acceleration rotation. We found that the time required for ORL mice to fall was longer than that of TBI mice in both uniform and uniform acceleration modes (Figure 2E), indicating that ORL can improve the motor function of TBI mice.

The above experimental results indicate that ORL treatment can significantly improve the degree of brain injury and neurological dysfunction after TBI.

### 2.2. Microglia-Dependent Mechanisms Underlie Orlistat’s Neuroprotective Effects

**Figure 3:**
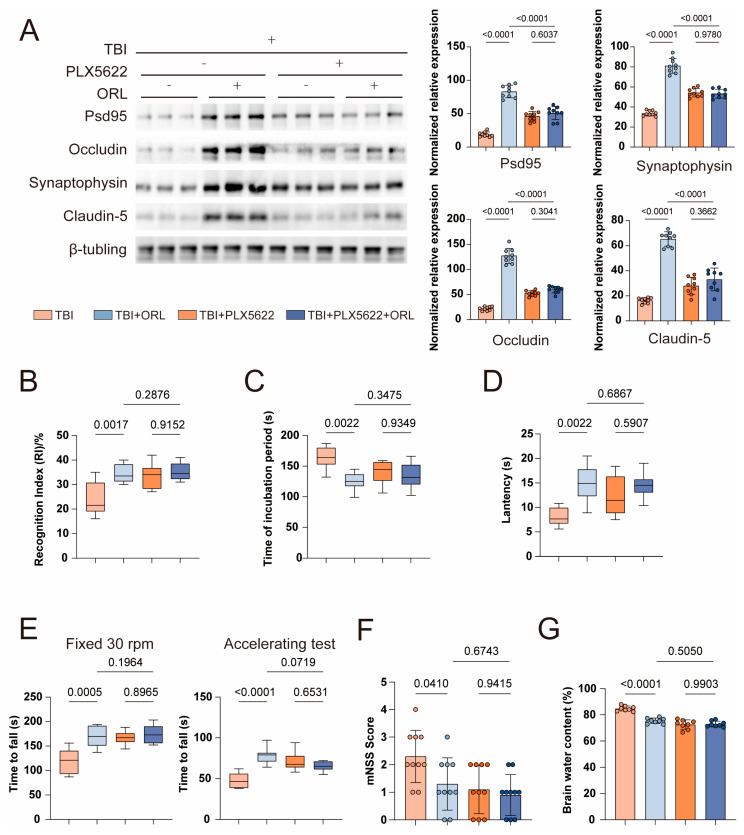
(**A**) Representative Western Blot images of Psd95, Occludin5, Synaptophysin and Caludin-5 in TBI mice after ORL treatment following the removal of intracranial microglia, *n* = 9. (**B**) Statistical chart of the results of new object recognition test conducted on TBI mice after ORL treatment following the removal of intracranial microglia, *n* = 10. (**C**) Statistical chart of the results of Barnes maze test conducted on TBI mice after ORL treatment following the removal of intracranial microglia, *n* = 10. (**D**) Statistical chart of the results of Barnes maze test conducted on TBI mice after ORL treatment following the removal of intracranial microglia, *n* = 10. (**E**) Statistical chart of the results of the rotarod test conducted on TBI mice after ORL treatment following the removal of intracranial microglia; the left chart is the result of the fixed-speed test at 30 rpm, and the right chart is the result of the acceleration test, *n* = 10. (**F**) mNSS score of TBI mice after ORL treatment following the removal of intracranial microglia, *n* = 10. (**G**) The water content of brain tissue in TBI mice after ORL treatment following the removal of intracranial microglia, *n* = 8.

Microglia are unique and the most important immune cells in the central nervous system, accounting for 5% to 20% of all glial cells. They play a significant role in the pathophysiological processes of the central nervous system [6]. To determine whether orlistat can exert protective functions through microglia, we administered PLX5622 to mice every day for seven days before modeling to eliminate microglia in the brain (Figure S3A) [13]. Subsequently, we conducted memory and motor function tests on the mice again. The results showed that under the treatment of TBI, after the original microglia were removed, the recognition frequency of new objects in ORL mice was no different from that in TBI mice treated by PLX5622 (Figure 3B and Figure S3B), and the performance of ORL mice in Barnes’ Maze and Water maze was not much different from that of TBI mice treated by PLX5622 (Figure 3C,D and Figure S3C,D). In the rotarod experiment, the time required for ORL mice to fall was close to that of TBI mice treated by PLX5622 (Figure 3E). 

Then, we conducted another assessment of the mNSS score and the degree of brain edema. After removing microglia, the mNSS score of ORL mice did not improve compared with TBI mice without microglia (Figure 3F), and the water content of the brain was also similar to that of TBI mice with PLX5622 (Figure 3G). Meanwhile, the detection of blood–brain barrier related proteins also indicated that there was no significant difference in their integrity compared with that of TBI mice with PLX5622 (Figure 3A).

These results indicate that orlistat mainly mediates its protective effect on the central nervous system in TBI by influencing intracranial microglia.

### 2.3. Orlistat Suppresses Microglial Activation and Neuroinflammation Post-TBI

**Figure 4:**
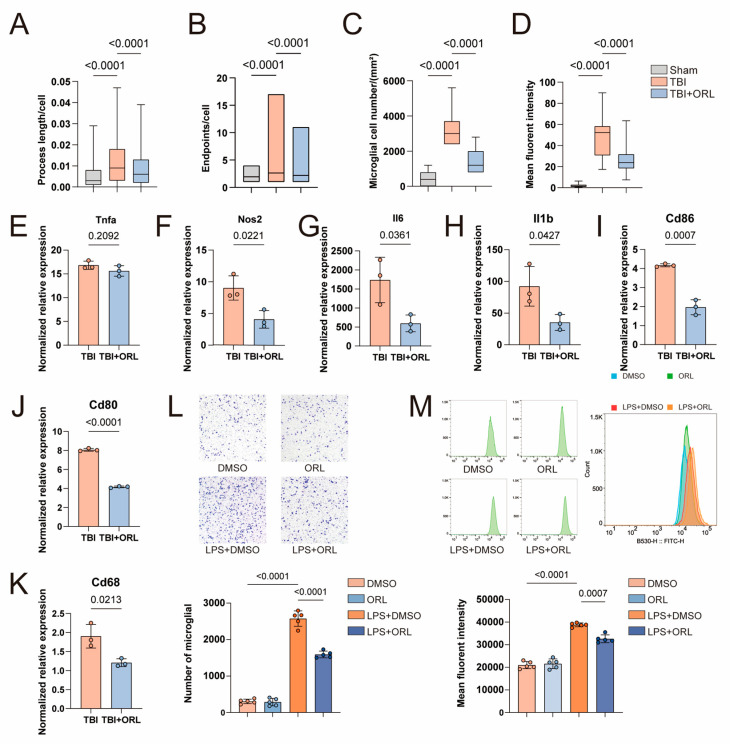
(**A**) The synaptic length of microglia infiltrating the injury site in TBI mice after ORL treatment. (**B**) The synaptic number of microglia infiltrating the injury site in TBI mice after ORL treatment. (**C**) The number of microglia infiltrating the injury site in TBI mice after ORL treatment. (**D**) The fluorescence intensity of CD68 at the lesion site in TBI mice after ORL treatment. (**E**) qPCR results of Tnfa in microglia of TBI mice after ORL treatment, *n* = 3. (**F**) qPCR results of Nos2 in microglia of TBI mice after ORL treatment, *n* = 3. (**G**) qPCR results of Il6 in microglia of TBI mice after ORL treatment, *n* = 3. (**H**) qPCR results of Il1b in microglia of TBI mice after ORL treatment, *n* = 3. (**I**) qPCR results of Cd86 in microglia of TBI mice after ORL treatment, *n* = 3. (**J**) qPCR results of Cd80 in microglia of TBI mice after ORL treatment, *n* = 3. (**K**) qPCR results of Cd68 in microglia of TBI mice after ORL treatment, *n* = 3. (**L**) The Transwell assay results of BV2 cells after administration of LPS and/or ORL, *n* = 5. (**M**) BV2 cells phagocytosed fluorescent beads after administration of LPS and/or ORL, and the intracellular fluorescence intensity was detected by flow cytometry, *n* = 5.

The previous research results pointed to the regulatory effect of orlistat on microglia. To further understand this effect, we performed immunofluorescence staining of Iba-1 and Cd68 on brain sections of mice with three days of TBI, where Iba-1 represents mature microglia and Cd68 is an early inflammatory indicator (Figure S4A,B). It can be seen that the microglia near the lesion after TBI are significantly activated, with an increase in local infiltration [14]. At the same time, the length and number of synapses both increase (Figure 4A–C), and the inflammatory response significantly increases (Figure 4D). However, ORL can inhibit this intense inflammatory response (Figure 4A–C). Then, in order to comprehensively detect the inflammatory response of microglia, we used antibodies of Cd45 combined with Cd11b to sort the intracranial microglia by flow cytometry and extract their RNA for RT-qPCR detection. We detected a total of 7 common inflammatory indicators of microglia, namely, Tnfa, Il1b, Il6, Nos2, Cd68, Cd80 and Cd86. Except for Tnfa, the other 6 inflammatory indicators all decreased to varying degrees after the use of ORL (Figure 4E–K).

Next, we studied the effect of ORL on microglia in vitro using BV2 and simulated the stimulation of TBI on microglia with LPS. In the Transwell experiment, LPS stimulation increased BV2 passing through the pinhole to the other side of the membrane, while the addition of ORL could reduce the BV2 infiltration caused by this LPS stimulation (Figure 4L). To test the phagocytic function of BV2, we added FITC-coupled standard beads to the culture medium. The stimulation of LPS significantly increased the number of microspheres phagocytized by BV2, and the intracellular fluorescence intensity was significantly enhanced. However, the increase in intracellular fluorescence intensity of BV2 treated with ORL was relatively small, indicating that it phagocytized fewer fluorescent beads (Figure 4M). This might suggest that after TBI, ORL can inhibit the phagocytic function of microglia, thereby reducing their excessive phagocytic pruning of neuronal synapses during normal inflammatory responses.

In conclusion, orlistat can reduce the inflammatory activation of microglia by TBI or LPS both in vivo and in vitro, thereby reducing their infiltration at the injury site and lowering secondary damage caused by excessive activation of microglia.

### 2.4. Lipoprotein Lipase (LPL) Emerges as a Key Target of Orlistat in Microglia

**Figure 5:**
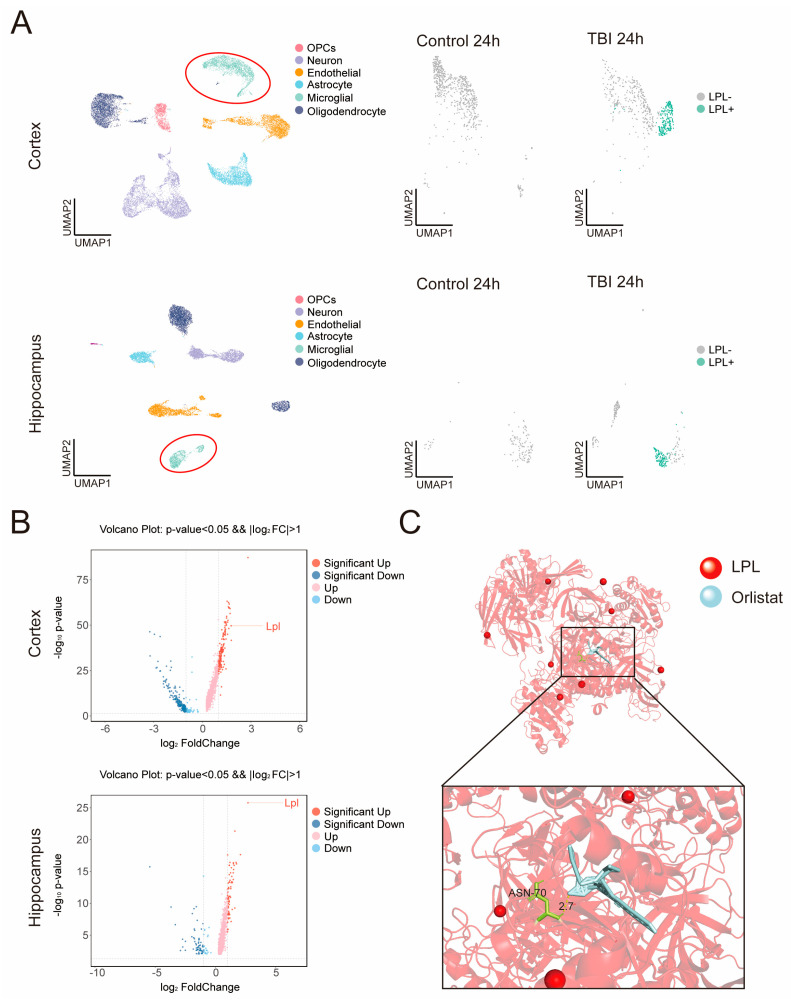
(**A**) The left side shows the single-cell sequencing clustering map of the cerebral cortex and hippocampal tissues of mice 24 h after TBI, and the right side shows that microglia can be further divided into LPL-positive cell populations and LPL-negative cell populations. (**B**) Volcano plot of differentially expressed genes between the LPL-positive cell population and the LPL-negative cell population. (**C**) Molecular docking results of LPL and Orlistat simulated by AutoDock (v 4.2.6); the red object represents LPL, and the cyan object represents Orlistat.

The inflammatory activation of microglia after TBI is a rapid and complex process, involving changes in multiple physiological and pathological pathways such as transcription, translation, and metabolism. To identify potential targets of orlistat acting on microglia, we retrieved single-cell sequencing data from the GEO database from the cortex and hippocampus 24 h after TBI to analyze the transcriptional changes of microglia 24 h after TBI [15,16]. Whether it is the cortex or the hippocampus, cells are mainly divided into neurons, astrocytes, oligodendrocytes, endothelial cells and microglia. We extracted the microglia separately and re-grouped them for analysis. Compared with the control group, a new microglia subpopulation emerged in the microglia 24 h after TBI, which might be highly correlated with the inflammatory activation of microglia after TBI. By analyzing the differential genes of this new subpopulation of microglia and other microglia, we found that in the cortex and hippocampus. All the newly added microglia expressed the LPL (lipoprotein lipase) gene highly (Figure 5A,B). LPL is a lipase expressed in the heart, muscle and adipose tissue, which has the activity of hydrolyzing triglycerides and receptor-mediated lipoprotein uptake [17]. The disorder of lipid metabolism is closely related to the inflammatory activation of microglia [18,19,20]. Among them, triglycerides, as the core lipid mediator, directly enter the skull through the damaged blood–brain barrier after TBI. After being taken up by microglia, they are decomposed into free fatty acids, which can directly activate the TLR4 receptor and thereby mediate the transformation of the downstream NF-κB pathway to a pro-inflammatory phenotype and release pro-inflammatory factors [20,21]. Meanwhile, triglyceride-derived fatty acids, such as stearic acid, can induce a burst of reactive oxygen species in microglia, activate the downstream NLRP3 inflammasome, and promote the maturation and release of IL-1β [22,23,24]. 

Next, we used AutoDock to predict the binding of LPL to orlistat and presented it through PyMol [25]. The docking results showed that the interaction site between orlistat and LPL was asparagine at position 70, connected by a hydrogen bond, with a bond energy of 2.7 KJ/mol (Figure 5C).

### 2.5. Transcriptomic and Metabolomic Profiling Reveals Orlistat’s Regulatory Effects on Microglial Function

**Figure 6:**
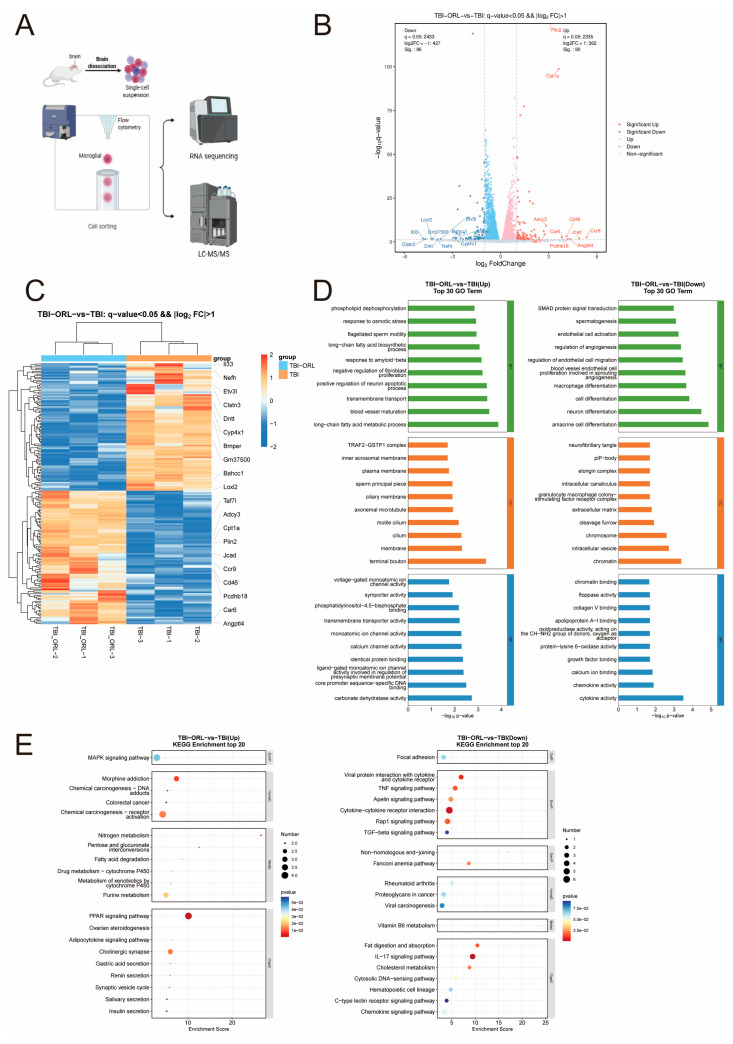
(**A**) Model diagram for transcriptome and metabolome detection of mouse microglia by flow cytometry sorting (created using BioRender.com). (**B**) Volcano plots of differentially expressed genes in microglia between the TBI-ORL group and the TBI group. (**C**) Heatmap of differentially expressed genes in microglia between the TBI-ORL group and the TBI group. (**D**) Gene Ontology (GO) analysis of differentially expressed genes in microglia between the TBI-ORL group and the TBI group. (**E**) Kyoto Encyclopedia of Genes and Genomes (KEGG) analysis of differentially expressed genes in microglia between the TBI-ORL group and the TBI group.

**Figure 7:**
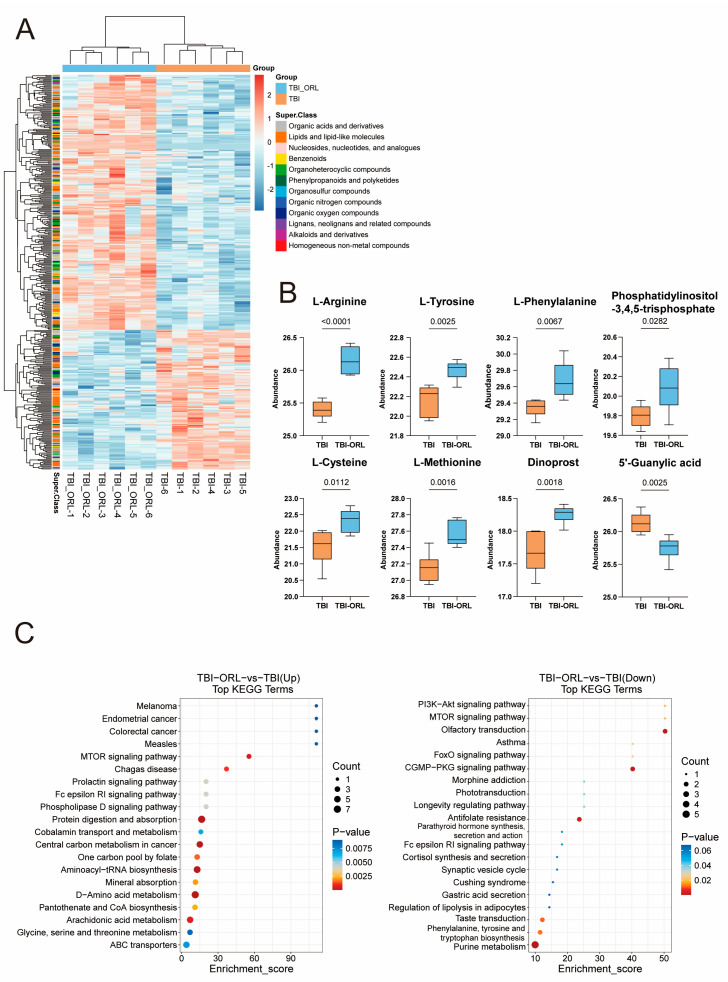
(**A**) Heatmap of differential metabolites in microglia between the TBI-ORL group and the TBI group. (**B**) Statistical chart of 8 representative differential metabolites in microglia between the TBI-ORL group and the TBI group. (**C**) Kyoto Encyclopedia of Genes and Genomes (KEGG) analysis of differential metabolites in microglia between the TBI-ORL group and the TBI group.

To further investigate the molecular mechanism by which orlistat inhibits microglial inflammation, we sorted microglia using antibodies against Cd11b and Cd45 and conducted transcriptome and metabolome assays (Figure 6A). The transcriptome results showed that the treatment of ORL induced the transcription of genes such as Plin2 and Cpt1a in microglia, inhibited the transcription of inflammation-related genes such as IL-33 (Figure 6B,C) [26,27,28,29,30], promoted the uptake and utilization of long-chain fatty acids, and inhibited the activation of endothelial cells (Figure 6D). In terms of pathways, ORL treatment activated pathways such as the PPAR pathway and MAPK pathway in microglia [31,32] while inhibiting pathways such as the IL-17 pathway and cholesterol metabolism (Figure 6E) [33,34,35,36]. The metabolomics detection results showed that after ORL treatment, substances such as L-arginine, PI3 and various amino acids in microglia were upregulated, AMP was downregulated, the mTOR signaling pathway was activated, the FoxO signaling pathway was inhibited (Figure 7A–C), and autophagy and related inflammatory responses were suppressed.

## 3. Discussion

Looking back at previous studies, most of them mainly focused on the targets and mechanisms of orlistat as a weight loss drug acting on adipocytes [37,38,39,40], as well as the preliminary exploration of its regulation of lipid metabolism in macrophages in the peripheral area [41,42]. Our research focuses on the potential protective effect of orlistat on the central nervous system, that is, its regulatory effect on microglia, the resident cells of the central immune system.

Orlistat, as a well-known weight loss drug, has relatively mild toxicological characteristics, but long-term use does indeed have certain side effects. Orlistat can reduce the absorption of dietary fat in the gastrointestinal tract, which may lead to uncomfortable oily stools or even fat loss. At the same time, the fermentation of some undigested fat in the intestines may cause abdominal distension or even fecal incontinence. These gastrointestinal reactions are usually dose-dependent and related to dietary structure. A low-fat diet can reduce the degree and frequency of the above-mentioned side effects. At the same time, due to the reduced absorption of lipids, the absorption of fat-soluble vitamins such as vitamin A and vitamin K will also be inhibited to a certain extent, which may cause corresponding vitamin deficiency conditions such as night blindness and bleeding. The blood drug concentrations of some other fat-soluble drugs may also decrease as a result. It is worth noting that orlistat has very rare but serious hepatotoxicity, as well as rare nephrotoxicity and allergic reactions. Overall, thanks to orlistat’s low systemic absorption rate, its long-term systemic toxicity risk is not high.

In the early stages of TBI, microglia are rapidly activated and release a substantial number of inflammatory factors. These high concentrations of inflammatory mediators may activate downstream inflammation-related signaling pathways, leading to secondary neuronal necrosis, further disruption of the blood–brain barrier, and exacerbation of cerebral edema. Furthermore, microglia serve as key initiators of the inflammatory “cascade effect” during the early phase of the disease. Evidence suggests that early depletion of microglia following TBI can significantly reduce the inflammatory response in the brains of experimental animals and alleviate cerebral edema. However, as the disease progresses, microglia gradually transition from a “pro-inflammatory phenotype” to an “anti-inflammatory phenotype.” In this later phase, their primary functions shift toward phagocytosis and tissue repair, including the clearance of cellular debris and apoptotic cells, as well as the secretion of neurotrophic factors that sup-port neuronal survival. In summary, microglia exhibit a dual role throughout the progression of TBI: during the acute phase, they contribute to inflammatory responses and tissue damage, whereas in the later stages, they facilitate repair processes and help maintain central nervous system homeostasis [1].

LPL is a lipoprotein lipase that is widely expressed in the heart, muscle and adipose tissue [17], and it has the dual functions of a triglyceride hydrolase and a receptor-mediated lipoprotein uptake ligand. Orlistat is a well-known irreversible inhibitor of pancreatic and gastric lipase, as well as a fatty acid synthase inhibitor [5], and it is commonly used to treat obesity and prevent atherosclerosis. The RCT of XENDOS indicates that orlistat has a more significant weight loss effect compared to placebo and can reduce the incidence of type 2 diabetes by 37% [40]. Long-term use can effectively control weight and improve lipid metabolism, and its long-term safety is guaranteed [40].

The polarization of microglia is closely related to lipid metabolism [43]. The accumulation of lipids can activate the inflammasome NLRP3 and promote the release of a series of inflammatory factors. Meanwhile, M1-type microglia focus on glycolysis, while M2-type microglia focus more on oxidative phosphorylation [18]. Changes in lipid metabolism also affect the polarization direction of microglia.

There are relatively few reports on LPL in TBI. In 2011, some researchers listed pioglitazone as one of the target genes of PPAP-γ in their study on secondary brain injury caused by experimental brain trauma [44]. LPL has been studied more frequently in other peripheral systems, but relatively less in the central nervous system. It may be involved in regulating energy balance and cognitive function in the brain. In the brain, the expression of LPL in neurons and vascular endothelial cells is relatively higher, and the region with the highest expression of LPL in the whole brain is the hippocampus. In the research of AD, mutations in LPL increase the risk of sporadic AD [45]. LPL can also bind to amyloid-β protein, promoting the uptake of it by astrocytes and influencing the pathophysiology of AD [45]. In cerebral ischemia, the upregulation of LPL expression may be involved in lipid recycling and neural remodeling [45].

Our research indicates that there is a subpopulation of microglia highly expressing LPL after TBI. LPL decomposes triglycerides into free fatty acids, providing lipid substrates for signal transduction and further intensifying the inflammatory activation of microglia. Orlistat inhibits this abnormal response and reduces the expression of pro-inflammatory cytokines. Metabolically, orlistat promotes fatty acid oxidation in microglia and inhibits cholesterol metabolism and IL-17 signaling, which not only alleviates the inflammatory response in the acute phase of TBI but may also be beneficial for treating chronic neurodegenerative diseases caused by TBI in the long term [33]. Additionally, orlistat activates the PPAR and MAPK pathways, promoting the transformation of microglia into the M2 type and facilitating tissue repair [46]. As a traditional weight loss drug, orlistat can lower lipid levels in circulation and may also help reduce the infiltration of lipids from the peripheral circulation through the damaged blood–brain barrier at the injury site, improving prognosis.

Although our study shows the effect of orlistat on microglia, it also suggests that orlistat has potential effects on other cells such as endothelial cells and neurons, which needs further exploration to determine its function in the entire central nervous system.

TBI, as a common central nervous system injury, has a high fatality and disability rate. However, there is still a lack of economically effective therapeutic drugs in clinical practice. Orlistat, as a mature and low-cost drug, clearly has potential in the treatment of TBI, thus providing an economical and effective alternative for TBI.

## 4. Conclusions

This study investigated the neuroprotective effects of orlistat (ORL) in a murine model of traumatic brain injury (TBI), with a particular emphasis on its modulation of microglial inflammatory responses. Behavioral, histological, and molecular biological analyses demonstrated that ORL mitigated neural damage induced by TBI. Following the depletion of brain microglia, the neuroprotective efficacy of ORL was significantly reduced, indicating that microglia may represent a key cellular target of ORL. In vitro experiments revealed that ORL suppressed microglial activation, as well as their phagocytic and migratory capacities. Single-cell sequencing analysis identified a marked upregulation of the lipoprotein lipase (LPL) gene in certain microglial populations following TBI. Molecular docking studies predicted a potential binding interaction between ORL and LPL, suggesting that ORL may directly inhibit LPL activity. Transcriptomic and metabolomic analyses further indicated that ORL exerts its protective effects by modulating microglial metabolic pathways and attenuating inflammatory responses.

In conclusion, orlistat enhances TBI outcomes by targeting microglial LPL and suppressing lipid metabolism-driven inflammatory responses. These findings provide a theoretical foundation for repurposing orlistat as a potential therapeutic agent for the treatment of TBI. However, this study only focused on the regulatory effect of orlistat on microglia, and its effects on other cells in the central nervous system remain unclear. More experiments are needed in the future to explore the overall effect of orlistat on the central nervous system and its possible role in the long-term prognosis of TBI.

## 5. Materials and Methods

### 5.1. Animals

All the animals used in the experiment and all the animal experiments conducted were approved by the Experimental Animal Ethics Committee of Huazhong University of Science and Technology. Male C57BL/10ScNJ mice (age, 6–8 weeks; weight, 17–22 g; at enrollment) were raised at the Experimental Animal Center of Tongji Science Building at Tongji Hospital. All mice were maintained a 12 h light/dark cycle in a controlled environment at 22 ± 3 °C and 60% relative humidity, and they received standard rodent nutrition and water. The total number of mice used in the study was about 300.

### 5.2. Animal Models

TBI was established using the controlled cortical impact (CCI) modification method as described above [8]. CCI was performed to create contusion in the brain. In brief, mice were anesthetized with 5% isoflurane at a flow rate of 0.5 L/min (in oxygen) and maintained with 1.5% isoflurane. All procedures were performed at room temperature. Aseptic surgical procedures were followed. A midline incision was made initially, and then a 3 mm^2^ area was exposed at the following specific location: 1 mm anterior to λ, 1 mm left to midline, and 1 mm posterior to bregma. In total, a 3 mm^2^ area of the skull was removed while avoiding damage to the dura mater. After the impact, local hemostasis was achieved using cotton balls, the scalp incision was sutured, and body temperature was maintained until anesthesia wore off. The CCI (YHCI99; Wuhan Yihong Technology, Wuhan, China) parameters were as follows: (1) deformation depth: 1 mm; (2) impact speed: 3.5 m/s; and (3) duration: 400 ms. Sham mice underwent craniotomy without CCI. The mice were placed on a heating pad at 36 °C to 37 °C. For orlistat treatment, orlistat (HY-B0218, MCE, China, 50 mg/kg) was intraperitoneally injected every 24 h starting 3 days before model creation, and it continued after model creation until sacrifice.

### 5.3. Microglia Clearance

PLX5622(HY-114153, MCE, China) was dissolved in DMSO and diluted with PBS to the final dose concentration. Starting from day one, mice were injected with PLX5622 (50 mg/kg) intraperitoneal injection, and the injection was repeated twice every 24 h for a total of 7 days, which could remove 90% of microglia in the body.

### 5.4. Animal Sampling

The mice were first anesthetized by induction with a 5% isoflurane–oxygen solution at a flow rate of 0.5 L/min and 2% isoflurane for maintenance. Then, all mice were euthanized by injecting ketamine (180 mg/kg) + xylazine (30 mg/kg). The hearts of mice were perfused with PBS buffer to replace their original blood. For histological experiments, the brains were fixed in 4% PFA at room temperature for 24 h and then treated with a gradient of sucrose concentrations for dehydration. The brain was embedded with paraffin and made into paraffin sections on microscope slides. For sorted microglia, samples were prepared into single-cell suspensions using the Gentle Enzymatic Implementation Kit for Adult Brain Tissue (RWD, Shenzhen, China), cells were labeled with Cd45, and Cd11b and microglia were isolated using a flow sorter.

### 5.5. The mNSS Score

On the third day after the adoption of TBI, the modified neurological severity score (mNSS) was used to evaluate neurological injury. This system assesses abnormalities in motor function and disorders in movement, sensation and response. The total score ranges from 0 to 18. The higher the score, the more severe the neuropathy. Three researchers independently conducted the assessment using a blind procedure, and the final score was determined as the average of the assessment.
mNSS ScoreExercise testing
➀ Tail lifting experiment
 Forelimb flexion1 Hindlimb flexion1 The head deviates from the vertical axis > 100° within 30 s1➁ Walking experiment
 Normal walking0 Unable to walk in a straight line1 Like hemiplegic lateral rotation2 Like hemiplegic lateral tilt3Sensory experiment
➀ Visual and tactile experiments
 Can mice avoid obstacles?1➁ Proprioceptive experiment
 Squeezing mouse claws to stimulate limb contraction1  Balance beam experiment
Stable balance posture0 Grasp the edge of the balance beam tightly1  Hold onto the balance beam tightly, with one limb hanging down from the balance beam2 Hold onto the balance beam tightly, with both limbs hanging down from the balance beam or rotate on the balance beam > 60 s3Attempted to balance on the balance beam but fell >40 s4 Attempted to balance on the balance beam but fell >20 s5 Falling, not attempting to balance on the balance beam < 20 s6Loss of reflexes and abnormal movements
 Shake your head when in contact with the external auditory canal1 Blink when cotton fibers lightly touch the cornea1 Has a motion response to the noise of fast-moving cardboard1 Epilepsy, myoclonus or dystonia1

### 5.6. Determination of Brain Water Content

After the mice were sacrificed, their brains were taken, and their wet weight was measured with an electronic scale. Then, the brain was wrapped in tin foil and placed in an oven to thoroughly dry the moisture. It was taken out, and its dry weight was measured. The water content of mouse brains was determined as follows: (Wet weight-Dry weight)/Wet weight.

### 5.7. Hematoxylin and Eosin (H&E) Staining

The sections were placed onto microscope slides, followed by deparaffinization in three sequential xylene changes and subsequent rehydration through a series of graded alcohol concentrations. First, the sample was stained with hematoxylin dye for 5 min and then rinsed with clean water for 10 min. Next, it was decomposed with hydrochloric acid ethanol for 3 to 5 s, rinsed with clean water for 10 min, then treated with anti-blue solution for 3 to 5 s, rinsed with clean water for 10 min, and then stained with eosin for 5 min. Finally, it was dehydrated with anhydrous ethanol for 5 min. After repeating three times, it was treated with xylene for 5 min to make it transparent, and the sheet was sealed with neutral gum.

### 5.8. Rotarod Test

On the third day of TBI, rotarod tests were conducted to evaluate the coordination ability, strength and balance ability of the mice. We placed the mice on a rotating stick. For the fixed speed test, the rotation speed was set to 30 rpm, and the maximum test time was set to 300 s. For the accelerating test, the initial rotation speed was set to 30 rpm and accelerated gradually. The delay of the decline was recorded, each mouse was tested once or twice, and the average of the two tests was analyzed.

### 5.9. Open-Field Test

Before the test began, all the mice were placed in the experimental environment for 1 h adaption. VisuTrack software (XR-VT, Xinruan, Shanghai, China) was used to pre-set the relevant parameters, and the animal identification numbers and dates were recorded. After the experiment started, the animal was put in the experiment box, and the experiment started. The duration of the experiment was 5 min. The area was then disinfected, and subsequent animals entered the laboratory from the same direction. Each mouse was tested twice.

### 5.10. Novel Object Recognition (NOR) Test

The mice were allowed to enter the laboratory to familiarize themselves with the environment the day before the experiment. After the training began, two identical blue bottles were placed in the center of the stadium. The mouse was placed on the stage with its back to the two objects. The number of detections of the two objects within five minutes and the total time were recorded. After the experiment, the mouse was removed, and the objects and the arena were cleaned and disinfected. One of the blue bottles was replaced with another red one and placed in the arena. After 1 h’s rest, the experiment was repeated. The number of contacts with the two objects (one of which was a new object) and the total search time were recorded for 5 min. After the experiment was over, the mice were put back into the cage. The calculation formula for the recognition index (RI) is RI = (time spent with a new object)/(time spent with a new object + time spent with an old object) × 100%. All the bottles used in the experiment were slightly larger in size than the mice.

### 5.11. Barnes Maze Test

Seven days after TBI modeling, the Barnes Maze test was used to evaluate the spatial memory function of mice. In order to adapt to the experimental environment more easily, the laboratory mice entered the laboratory one day before the experiment. This experiment lasted for a total of 7 days, including 6 days of training and the exam on the last day. When the test began, the mice were placed in the central area of the maze and could explore freely. Environmental noise was used as a stimulus to guide the mice into the escape tunnel. The experiment ended when the mice entered the escape tunnel or after a 5 min exploration. During the training period, if the mice failed to find the escape tunnel within 5 min, they were quietly placed in the tunnel and stayed there for 60 s. On this day, we carefully recorded the incubation period of the mice before they found the correct hole. These data were analyzed to evaluate the learning and memory abilities of the mice. At the end of each experiment, we thoroughly cleaned all the equipment.

### 5.12. Morris Water Maze (MWM)

Spatial learning and memory were tested from the 7th postoperative day using the MWM (XR-XM101, Xinruan, Shanghai, China). The installation included a swimming pool (diameter 1.5 m, height 60 cm) filled with water (temperature 21–25 °C). The mice were trained to search for a platform immersed to a depth of 1.5 cm using a marker around the side walls. During the 6 days of training, the platform was placed in the third quadrant of the test site. During the daily timed tests, all mice were given 60 s to find the platform and 30 s to stay there. At the start of each trial, the mice were randomly placed in one of three quadrants (first, second and fourth). The software wmt-100 Morris (Chengdu Taimeng technology, Chengdu, China) was used to record the average latency, distance traveled and swimming speed in front of the positioning platform of each time trial on the day of the test. On the seventh day, a space exploration assessment was conducted, the platform was removed, and the mice were given 60 s to locate the area where the platform was located. The time spent by the mouse in the target quadrant and the number of passes through the previous platform position were recorded.

### 5.13. Sucrose Preference Test

Before the experiment, mice performed adaptability training on 1% (*w*/*v*) sucrose solution for 72 h: Two bottles containing 1% sucrose solution were placed in each cage. After 24 h, pure water was used instead of 1% sucrose solution in one of the bottles. After 24 h, the mice were banned from the water and fasted for 24 h. A sugar water preference experiment was conducted. During the experiment, mice could drink 2 bottles of water freely: 1 bottle contained 1% sucrose solution, and the other bottle contained pure water. The positions of the two bottles were frequently swapped (left or right). After 12 h, the remaining liquid was measured to determine the consumption of sucrose solution and pure water by the mouse within 12 h. Sugar water preference rate (%) = sugar water consumption/(sugar water consumption + pure water consumption) × 100%.

### 5.14. ELISA

After the mice were sacrificed, their brains were taken. First, 30 mg of brain tissue was placed in a grinding tube, and 500 μL of DMEM was added. The tissue was manually ground on ice and then filtered through a 40 μm filter screen. The filtrate was centrifuged at 10,000× *g* for 20 min, and the supernatant was taken for subsequent testing.

Prepare the samples and standards according to the instructions of the Elisa kit (Abclonal, Wuhan, China). Add 350 μL of washing buffer to each well, let it stand for 40 s, and then discard the liquid. Repeat this process three times. Then, add 100 μL of the sample and the standard to the corresponding Wells. Seal the wells with a sealing film, and incubate at 37 °C for 2 h. Discard the liquid in each well, and wash it three times again with the washing buffer as mentioned earlier. Then add 100 μL of biotinylated antibody working solution to each well, seal the wells again, and incubate at 37 °C for 1 h. Discard the liquid in the wells. Wash the wells three times again with the washing buffer as mentioned earlier. Then add 100 μL of streptavidin-HRP solution to each well, seal the wells, and incubate at 37 °C for 30 min. Discard the liquid in each well, and wash it three times with the washing buffer as mentioned earlier. Then add 100 μL of TMB substrate to each well, and incubate at 37 °C in the dark for 15–20 min. Finally, add 50 μL of stop solution to each well. The 450 nmOD value was determined using a microplate reader.

### 5.15. Evans Blue Blood–Brain Barrier

On the third day after the TBI model was established in the experimental animals, the mice were placed in a tail vein injection device, and 0.5% Evans Blue Stain (2 mL/kg) was injected through the tail vein. After circulating the dye for one hour, the animals were euthanized, and the brain tissues were separated. After cardiac perfusion with PBS, the brains of the mice were collected and weighed. PBS buffer was added to the sample, and then it was homogenized in a glass tube and centrifuged at 12,000 rpm at 4 °C for 5 min. It was incubated at night at 4 °C, hydrogen peroxide was collected, and 50% trichloroacetic acid was added. The sample solution was examined with a 610 nm spectrometer. Quantification was carried out using standardized tissue weight curves (per microgram). When shooting, the brain tissue was fixed in 4% PFA for 24 h, then the brain tissue was cut into 2–3 mm thin slices with a blade, and pictures were taken.

### 5.16. Nissl Body Staining

The sections were placed onto microscope slides, followed by deparaffinization in three sequential xylene changes and subsequent rehydration through a series of graded alcohol concentrations. The slices were soaked in Nissl’s dye solution (G1036, Servicebio, China), stained at room temperature for 10 min, and then washed 3 times with distilled water, followed by dehydration with 95% ethanol for 2 min, which was repeated twice; finally, they underwent transparent treatment for 5 min with xylene and were sealed with neutral gum.

### 5.17. Cell Culture

BV2 cells were acquired from the American Type Culture Collection (ATCC, USA). All cells were cultured in Dulbecco’s Modified Eagle Medium (DMEM, G4515, Servicebio), supplemented with 10% fetal bovine serum (FBS, 10099141C, Gibco, USA) and 1% penicillin–streptomycin (G4003, Servicebio) at 37 °C with 5% CO_2_. For LPS treatment, LPS (HY-D1056, MCE, China, 500 ng/mL) was added to the culture medium and cultured for 24 h. For orlistat treatment, orlistat (HY-B0218, MCE, China, 50 μM) was added to the culture medium and cultured for 24 h.

### 5.18. Transwell Assay

The Transwell experiment was used to analyze cell migration. The upper cavity was filled with culture that did not contain fetal serum, while the lower cavity was filled with culture that contained 10% fetal serum. Then, LPS and/or orlistat-treated BV2 cells (5 × 10^4^/chamber) were inoculated into the upper chamber and incubated at 37 °C for 24 h. Then the top of the film was fixed with 4% paraformaldehyde at room temperature for 30 min and wiped with a cotton swab. A crystal purple solution of cells moved into the lower cavity (G1014, Servicebio) for 30 min at room temperature. Finally, the cells in the four random fields of each well were counted at a magnification of 20 times using a bright-field microscope to calculate the mobility of microglia.

### 5.19. Flow Cytometry

BV2 cells treated with LPS and/or orlistat were collected after adding 2 μL fluorescent beads (Cat#17147-5, Polysciences, USA) to the culture medium and culturing for 24 h. Cells were washed with PBS buffer 3 times, and then single-cell samples were detected by CytoFLEX (Beckman, USA) and analyzed by FlowJo v10.8.1 for the following statistical analysis.

### 5.20. RNA Isolation and Library Preparation

Total RNA was extracted using the TRIzol reagent (Thermo Fisher Scientific, USA) according to the manufacturer’s protocol. RNA purity and quantification were evaluated using the NanoDrop 2000 spectrophotometer (Thermo Fisher Scientific, USA). RNA integrity was assessed using the Agilent 2100 Bioanalyzer (Agilent Technologies, Santa Clara, CA, USA). Then libraries were constructed using VAHTS Universal V10 RNA-seq Library Prep Kit (Premixed Version, Vazyme, China) according to the manufacturer’s instructions. The transcriptome sequencing and analysis were conducted by OE Biotech Co., Ltd. (Shanghai, China).

### 5.21. Western Blot

After the experimental animals were sacrificed and perfusion was performed, their brain tissues were isolated. The brain tissue around the cortical injury area of the left cerebral hemisphere was resected and added to ripa decomposition buffer (Ripa: phosphorylated protease inhibitor A:B:PMSF = 100:1:1:1, G2007, Servicebio, China). Then an ultrasonic device was used to homogenize the tissue. Telocentric separation was carried out at 13,000× *g* at 4 °C for 30 min, and the supernatant was extracted from animal tissues as a protein sample. The protein concentration of the samples was detected and adjusted at a wavelength of 570 nm using a BCA protein quantification detection kit (G2026, Servicebio, China) and a microplate reader (Infinite F50, Tecan Group Ltd., Swiss). First, 5× SDS-PAGE protein sample loading buffer (G2075, Servicebio) was added to the sample, and it was heated in a 100 °C water bath for 10 min. After separating the proteins in equal amounts (10 µg/lane) with 6%, 8%, 10% and 12% SDS-PAGE gels, they were transferred to Immobilon^®^-P PVDF membranes (IPVH00010, MilliporeSigma, Germany). After 2 h of blocking with 5% BSA at room temperature, the membrane was immersed in the primary antibody solution and incubated at 4 °C overnight. Then, the membrane was washed with TBST buffer, transferred to the secondary antibody solution, and incubated at room temperature for 2 h. The protein bands were visualized by BeyoECL Plus (P0018S, Beyotime, China) and gel imaging systems (GeneGnome XRQ, Syngene, UK). The gray values of the protein bands were statistically analyzed using ImageJ 1.8.0 software, and, finally, the expression levels of the target proteins were normalized to the expression levels of the internal reference proteins.

### 5.22. RT-qPCR

According to the manufacturer’s instructions, total RNA was extracted from cells using TRIzol reagent (Thermo Fisher Scientific). RNA concentration and purity were assessed using an ultra-micro-spectrophotometer (KAIAO K5800, China). Subsequently, mRNA was reverse-transcribed into cDNA using the HiScript II Q RT SuperMix for qPCR (R223-01, Vazyme, China). Real-time quantitative PCR (RT-qPCR) was carried out using ChamQ SYBR qPCR Master Mix (Q341-02, Vazyme, China) on a QuantStudio™ 1 Plus system (Thermo Fisher Scientific) under the following thermal cycling conditions: one initial denaturation step at 95 °C for 30 s, followed by 40 cycles of 95 °C for 10 s and 60 °C for 30 s. A final melt curve analysis was conducted from 60 °C to 95 °C to confirm amplification specificity. The relative expression level of the target gene was normalized to the internal reference gene GAPDH and calculated using the 2^−ΔΔCq^ method, where ΔCq = Cq (target gene) − Cq (GAPDH), and ΔΔCq = ΔCq (target gene) − ΔCq (control). The gene expression fold change of the experimental group was compared with that of the control group.

### 5.23. Immunofluorescence Staining

The sections were placed onto microscope slides, followed by deparaffinization in three sequential xylene changes and subsequent rehydration through a series of graded alcohol concentrations. Sections were repaired using Tris-EDTA (pH = 8.0) antigen retrieval solution (G1206, Servicebio, China) at 95 °C for 20 min. Subsequently, primary antibodies were diluted in 5% BSA solution, were added dropwise to the tissues, and incubated at 4 °C overnight. The next day, the slides were washed with PBS buffer for 10 min, which was repeated three times, and then the secondary antibody was incubated at room temperature for 2 h. Finally, the slides were sealed with coverslips using fluorescence anti-fade reagent containing Dapi. Images were captured using a fluorescence microscope (catalog no. CKX53; Olympus Corporation, Germany). ImageJ 1.8.0 software was used for data analysis.

### 5.24. RNA Sequencing and Differentially Expressed Genes Analysis

The libraries were sequenced on the Illumina NovaSeq 6000 platform to generate 150 bp paired-end reads. Approximately 25 M raw reads were obtained per sample. The raw reads in FASTQ format were initially processed using FastP (v 0.20.1) to remove low-quality sequences and adapter contaminants, resulting in high-quality clean reads. On average, approximately 24 M clean reads per sample were retained and subsequently used for downstream analyses. The clean reads were aligned to the reference genome using HISAT2 (v 2.1.0) for accurate mapping. Gene expression levels were quantified as FPKM (Fragments Per Kilobase of transcript per Million mapped reads), and gene-level read counts were calculated using HTSeq-count. Principal component analysis (PCA) was performed using R (v 3.2.0) to evaluate biological reproducibility across samples.

Differential expression analysis was conducted using DESeq2 (v 1.22.2). Genes exhibiting a Q value < 0.05 and an absolute fold change ≥ 2 (i.e., fold change > 2 or <0.5) were identified as significantly differentially expressed genes (DEGs). Hierarchical clustering analysis of DEGs was carried out using R (v 3.2.0) to visualize gene expression patterns across different experimental groups and samples. A radar plot depicting the top 30 differentially expressed genes was generated using the R package ggradar to illustrate the expression profiles of up-regulated and down-regulated DEGs.

Functional enrichment analyses, including Gene Ontology (GO), Kyoto Encyclopedia of Genes and Genomes (KEGG), Reactome, and WikiPathways, were performed based on the hypergeometric distribution using R (v 3.2.0) to identify significantly enriched biological terms and pathways. Visualization of the results was achieved through bar plots, chord diagrams, and bubble plots generated using R (v 3.2.0).

Gene Set Enrichment Analysis (GSEA) was carried out using GSEA software (v 4.3.0) with a predefined gene set. Genes were ranked based on their differential expression levels between the two sample groups. The analysis assessed whether the predefined gene set was significantly enriched at either the top or bottom of the ranked gene list, indicating coordinated expression changes within the gene set.

### 5.25. LC-MS/MS

The experimental procedure is as follows: First, transfer the sample to a 1.5 mL EP tube in two steps using a total of 600 μL of pre-cooled methanol–water (*v*/*v* = 4:1, containing mixed internal standards) and thoroughly disperse it by pipetting. Subsequently, perform ice-bath sonication (1620 W, 10 min, 6 s on/4 s off), followed by ice-water bath-assisted extraction for 20 min and overnight incubation at −40 °C. The next day, centrifuge the sample for 10 min (12,000 rpm, 4 °C), transfer 400 μL of the supernatant to an LC-MS vial, and dry it under a gentle stream of nitrogen or vacuum. Reconstitute the dried sample with 300 μL of methanol–water (*v*/*v* = 1:4), vortex for 1 min, sonicate for 10 min, and incubate again at −40 °C overnight. Finally, centrifuge for 20 min (12,000 rpm, 4 °C), and transfer 150 μL of the supernatant to an LC-MS vial with an insert for analysis. Quality control (QC) samples are created by extracting liquid from all samples.

The analytical instrument for this experiment was a liquid–mass spectrometry system composed of Waters ACQUITY UPLC I-Class plus/Thermo QE (Waters/Thermo, USA) ultra-high-performance liquid phase tandem high-resolution mass spectrometer.

### 5.26. Molecular Docking

For the target of orlistat on LPL, the SDF file of orlistat was obtained from PubChem, the protein structure of LPL was obtained from the PDB database, and AutoDock was used to simulate the docking process. The docking results are displayed by PyMol (v 3.1.5.1).

### 5.27. Statistical Method

Statistical analysis of data was performed using IBM SPSS Statistics version 25.0 (IBM Software Group, Chicago, IL, USA). For data conforming to a normal distribution, either unpaired or paired two-tailed Student’s *t*-test and one-way and two-way analyses of variance (ANOVAs) were employed. Tukey’s test was utilized for correcting multiple comparisons. For data not meeting the assumption of a normal distribution, the Mann–Whitney U test was applied for comparisons between two groups, while the Kruskal–Wallis test was used for comparisons among multiple groups. A *p*-value less than 0.05 was considered statistically significant.

## Data Availability

The standardized datasets were uploaded in the Supplementary Files, and any other datasets used and analyzed in the current study are available from the corresponding author upon reasonable request.

## Supplementary Materials

The following supporting information can be downloaded at: https://www.mdpi.com/article/10.3390/cells14181469/s1, Figure S1: (A) The concentration of IL-1β in the brain of TBI mice after different doses of ORL treatment, *n* = 5. (B) The concentration of IL-6 in the brain of TBI mice after different doses of ORL treatment, *n* = 5. (C) The concentration of TNF-α in the brain of TBI mice after different doses of ORL treatment, *n* = 5. (D) The mNSS score of TBI mice after different doses of ORL treatment, *n* = 5. (E) Representative images of MBP and Neun at the injury site in TBI mice after ORL treatment were taken to assess the integrity of the myelin sheath and the number of neurons near the injury site (red = Mbp, green = Neun, blue = Dapi, scale bar = 50 μm). On the right, the upper chart shows the statistical results of myelin integrity, and the lower chart shows the statistical results of live neurons. Figure S2: (A) Representative figure of the open-field test conducted on TBI mice after ORL treatment. The right shows the experimental strategies that the mice were tested on 72 h after TBI. (B) Representative figure of the new object recognition test conducted on TBI mice after ORL treatment. The right shows the experimental strategies that the mice were tested on 72 h after TBI. (C) Representative figure of the Barnes maze test conducted on TBI mice after ORL treatment. The right shows the experimental strategies that the mice were trained on for 6 d and tested on 7 d after TBI. (D) Representative figure of the Morris water maze conducted on TBI mice after ORL treatment. The right shows the experimental strategies that the mice were trained on for 6 d and tested on 7 d after TBI. Figure S3: (A) Representative images of Iba-1 in mice after PLX5622 treatment to remove the intracranial microglia (green = Iba-1, blue = Dapi, scale bar = 20 μm). The right shows a statistical chart of the number of microglia (Iba-1-positive cells), *n* = 3. (B) Representative figure of the new object recognition test conducted on TBI mice after ORL treatment following the removal of intracranial microglia. (C) Representative figure of the Barnes maze test conducted on TBI mice after ORL treatment following the removal of intracranial microglia. (D) Representative figure of the Morris water maze conducted on TBI mice after ORL treatment following the removal of intracranial microglia. Figure S4: (A) Representative images of Cd68 and Iba-1 at the injury site in TBI mice after ORL treatment (red = Cd68, green = Iba-1, blue = Dapi, scale bar = 50 μm). (B) Quantitative statistics of fluorescence co-localization of Iba-1 and Cd68.

## Abbreviations

TBI	Traumatic brain injury
ORL	Orlistat
LPL	Lipoprotein lipase
mNSS	Mahmood’s method neurological severity score
BBB	Blood-brain barrier
WB	Western blot
RT-qPCR	Reverse Transcription Quantitative Polymerase Chain Reaction
LPS	Lipopolysaccharide
GEO	Gene Expression Omnibus
TLR4	Toll-like receptor 4
NF-κB	Nuclear factor kappa-B
NLRP3	NOD-like receptor thermal protein domain associated protein 3
PPAR	Peroxisome proliferators-activated receptors
MAPK	Mitogen-activated protein kinase
mTOR	Mammalian target of rapamycin
FoxO	Forkhead box O
RCT	Randomized controlled trial
AD	Alzheimer’s disease
CCI	Controlled cortical impact
NOR	Novel object recognition
MWM	Morris water maze
ELISA	Enzyme-linked immunosorbnent assay
GO	Gene Ontology
KEGG	Kyoto Encyclopedia of Genes and Genomes
GSEA	Gene Set Enrichment Analysis
LC-MS/MS	Liquid Chromatography-Tandem Mass Spectrometry
LC-MS	Liquid Chromatography-Mass Spectrometry

## References

[1] Shao F., Wang X., Wu H., Wu Q., Zhang J. (2022). Microglia and Neuroinflammation: Crucial Pathological Mechanisms in Traumatic Brain Injury-Induced Neurodegeneration. Front. Aging Neurosci..

[2] Bolte A.C., Lukens J.R. (2021). Neuroimmune cleanup crews in brain injury. Trends Immunol..

[3] Capizzi A., Woo J., Verduzco-Gutierrez M. (2020). Traumatic Brain Injury: An Overview of Epidemiology, Pathophysiology, and Medical Management. Med. Clin. N. Am..

[4] Corps K.N., Roth T.L., McGavern D.B. (2015). Inflammation and neuroprotection in traumatic brain injury. JAMA Neurol..

[5] McNeely W., Benfield P. (1998). Orlistat. Drugs.

[6] Wolf S.A., Boddeke H.W., Kettenmann H. (2017). Microglia in Physiology and Disease. Annu. Rev. Physiol..

[7] Wei W., Lattau S.S.J., Xin W., Pan Y., Tatenhorst L., Zhang L., Graf I., Kuang Y., Zheng X., Hao Z. (2024). Dynamic Brain Lipid Profiles Modulate Microglial Lipid Droplet Accumulation and Inflammation Under Ischemic Conditions in Mice. Adv. Sci..

[8] He X., Huang Y., Liu Y., Zhang X., Wang Q., Liu Y., Ma X., Long X., Ruan Y., Lei H. (2023). Astrocyte-derived exosomal lncRNA 4933431K23Rik modulates microglial phenotype and improves post-traumatic recovery via SMAD7 regulation. Mol. Ther..

[9] Othman Z.A., Zakaria Z., Suleiman J.B., Ghazali W.S.W., Mohamed M. (2021). Anti-Atherogenic Effects of Orlistat on Obesity-Induced Vascular Oxidative Stress Rat Model. Antioxidants.

[10] Xu Y., He Q., Wang M., Wang X., Gong F., Bai L., Zhang J., Wang W. (2019). Quantifying blood-brain-barrier leakage using a combination of evans blue and high molecular weight FITC-Dextran. J. Neurosci. Methods.

[11] Jamjoom A.A.B., Rhodes J., Andrews P.J.D., Grant S.G.N. (2021). The synapse in traumatic brain injury. Brain.

[12] Obermeier B., Daneman R., Ransohoff R.M. (2013). Development, maintenance and disruption of the blood-brain barrier. Nat. Med..

[13] Badimon A., Strasburger H.J., Ayata P., Chen X., Nair A., Ikegami A., Hwang P., Chan A.T., Graves S.M., Uweru J.O. (2020). Negative feedback control of neuronal activity by microglia. Nature.

[14] Alam A., Thelin E.P., Tajsic T., Khan D.Z., Khellaf A., Patani R., Helmy A. (2020). Cellular infiltration in traumatic brain injury. J. Neuroinflamm..

[15] Arneson D., Zhang G., Ahn I.S., Ying Z., Diamante G., Cely I., Palafox-Sanchez V., Gomez-Pinilla F., Yang X. (2022). Systems spatiotemporal dynamics of traumatic brain injury at single-cell resolution reveals humanin as a therapeutic target. Cell. Mol. Life Sci..

[16] Zhang G., Diamante G., Ahn I.S., Palafox-Sanchez V., Cheng J., Cheng M., Ying Z., Wang S.S., Abuhanna K.D., Phi N. (2024). Thyroid hormone T4 mitigates traumatic brain injury in mice by dynamically remodeling cell type specific genes, pathways, and networks in hippocampus and frontal cortex. Biochim. Biophys. Acta Mol. Basis Dis..

[17] Sagoo G.S., Tatt I., Salanti G., Butterworth A.S., Sarwar N., van Maarle M., Jukema J.W., Wiman B., Kastelein J.J., Bennet A.M. (2008). Seven lipoprotein lipase gene polymorphisms, lipid fractions, and coronary disease: A HuGE association review and meta-analysis. Am. J. Epidemiol..

[18] Orihuela R., McPherson C.A., Harry G.J. (2016). Microglial M1/M2 polarization and metabolic states. Br. J. Pharmacol..

[19] Chausse B., Kakimoto P.A., Kann O. (2021). Microglia and lipids: How metabolism controls brain innate immunity. Semin. Cell Dev. Biol..

[20] Haney M.S., Palovics R., Munson C.N., Long C., Johansson P.K., Yip O., Dong W., Rawat E., West E., Schlachetzki J.C.M. (2024). APOE4/4 is linked to damaging lipid droplets in Alzheimer’s disease microglia. Nature.

[21] Yao X., Yang C., Jia X., Yu Z., Wang C., Zhao J., Chen Y., Xie B., Zhuang H., Sun C. (2024). High-fat diet consumption promotes adolescent neurobehavioral abnormalities and hippocampal structural alterations via microglial overactivation accompanied by an elevated serum free fatty acid concentration. Brain Behav. Immun..

[22] Heneka M.T., Kummer M.P., Stutz A., Delekate A., Schwartz S., Vieira-Saecker A., Griep A., Axt D., Remus A., Tzeng T.C. (2013). NLRP3 is activated in Alzheimer’s disease and contributes to pathology in APP/PS1 mice. Nature.

[23] Terzioglu G., Young-Pearse T.L. (2023). Microglial function, INPP5D/SHIP1 signaling, and NLRP3 inflammasome activation: Implications for Alzheimer’s disease. Mol. Neurodegener..

[24] Han X., Xu T., Fang Q., Zhang H., Yue L., Hu G., Sun L. (2021). Quercetin hinders microglial activation to alleviate neurotoxicity via the interplay between NLRP3 inflammasome and mitophagy. Redox Biol..

[25] Forli S., Huey R., Pique M.E., Sanner M.F., Goodsell D.S., Olson A.J. (2016). Computational protein-ligand docking and virtual drug screening with the AutoDock suite. Nat. Protoc..

[26] Xu D., Zhuang S., Chen H., Jiang M., Jiang P., Wang Q., Wang X., Chen R., Tang H., Tang L. (2024). IL-33 regulates adipogenesis via Wnt/beta-catenin/PPAR-gamma signaling pathway in preadipocytes. J. Transl. Med..

[27] Vainchtein I.D., Chin G., Cho F.S., Kelley K.W., Miller J.G., Chien E.C., Liddelow S.A., Nguyen P.T., Nakao-Inoue H., Dorman L.C. (2018). Astrocyte-derived interleukin-33 promotes microglial synapse engulfment and neural circuit development. Science.

[28] Nguyen P.T., Dorman L.C., Pan S., Vainchtein I.D., Han R.T., Nakao-Inoue H., Taloma S.E., Barron J.J., Molofsky A.B., Kheirbek M.A. (2020). Microglial Remodeling of the Extracellular Matrix Promotes Synapse Plasticity. Cell.

[29] He D., Xu H., Zhang H., Tang R., Lan Y., Xing R., Li S., Christian E., Hou Y., Lorello P. (2022). Disruption of the IL-33-ST2-AKT signaling axis impairs neurodevelopment by inhibiting microglial metabolic adaptation and phagocytic function. Immunity.

[30] Wang M., Dufort C., Du Z., Shi R., Xu F., Huang Z., Sigler A.R., Leak R.K., Hu X. (2024). IL-33/ST2 signaling in monocyte-derived macrophages maintains blood-brain barrier integrity and restricts infarctions early after ischemic stroke. J. Neuroinflamm..

[31] Jamwal S., Blackburn J.K., Elsworth J.D. (2021). PPARgamma/PGC1alpha signaling as a potential therapeutic target for mitochondrial biogenesis in neurodegenerative disorders. Pharmacol. Ther..

[32] Iroegbu J.D., Ijomone O.K., Femi-Akinlosotu O.M., Ijomone O.M. (2021). ERK/MAPK signalling in the developing brain: Perturbations and consequences. Neurosci. Biobehav. Rev..

[33] Waisman A., Hauptmann J., Regen T. (2015). The role of IL-17 in CNS diseases. Acta Neuropathol..

[34] Chen H., Tang X., Li J., Hu B., Yang W., Zhan M., Ma T., Xu S. (2022). IL-17 crosses the blood-brain barrier to trigger neuroinflammation: A novel mechanism in nitroglycerin-induced chronic migraine. J. Headache Pain..

[35] Tcw J., Qian L., Pipalia N.H., Chao M.J., Liang S.A., Shi Y., Jain B.R., Bertelsen S.E., Kapoor M., Marcora E. (2022). Cholesterol and matrisome pathways dysregulated in astrocytes and microglia. Cell.

[36] Saher G. (2023). Cholesterol Metabolism in Aging and Age-Related Disorders. Annu. Rev. Neurosci..

[37] Tak Y.J., Lee S.Y. (2021). Long-Term Efficacy and Safety of Anti-Obesity Treatment: Where Do We Stand?. Curr. Obes. Rep..

[38] Foxcroft D.R., Milne R. (2000). Orlistat for the treatment of obesity: Rapid review and cost-effectiveness model. Obes. Rev..

[39] Feng X., Lin Y., Zhuo S., Dong Z., Shao C., Ye J., Zhong B. (2023). Treatment of obesity and metabolic-associated fatty liver disease with a diet or orlistat: A randomized controlled trial. Am. J. Clin. Nutr..

[40] Torgerson J.S., Hauptman J., Boldrin M.N., Sjostrom L. (2004). XENical in the prevention of diabetes in obese subjects (XENDOS) study: A randomized study of orlistat as an adjunct to lifestyle changes for the prevention of type 2 diabetes in obese patients. Diabetes Care.

[41] Park Y.J., Gil T.Y., Jin B.R., Cha Y.Y., An H.J. (2023). Apocynin alleviates weight gain and obesity-induced adipose tissue inflammation in high-fat diet-fed C57BL/6 mice. Phytother. Res..

[42] Kant S., Kumar A., Singh S.M. (2013). Myelopoietic efficacy of orlistat in murine hosts bearing T cell lymphoma: Implication in macrophage differentiation and activation. PLoS ONE.

[43] Hu X., Leak R.K., Shi Y., Suenaga J., Gao Y., Zheng P., Chen J. (2015). Microglial and macrophage polarization-new prospects for brain repair. Nat. Rev. Neurol..

[44] Thal S.C., Heinemann M., Luh C., Pieter D., Werner C., Engelhard K. (2011). Pioglitazone reduces secondary brain damage after experimental brain trauma by PPAR-gamma-independent mechanisms. J. Neurotrauma.

[45] Wang H., Eckel R.H. (2012). Lipoprotein lipase in the brain and nervous system. Annu. Rev. Nutr..

[46] Thakur S., Dhapola R., Sarma P., Medhi B., Reddy D.H. (2023). Neuroinflammation in Alzheimer’s Disease: Current Progress in Molecular Signaling and Therapeutics. Inflammation.

