# Supplementary figures and images for: Orlistat Confers Neuroprotection in Traumatic Brain Injury by Modulating Microglial Lipid Metabolism
Source: Cells. 2025 Sep 19;14(18):1469. doi: 10.3390/cells14181469 (PMC12468502; doi:10.3390/cells14181469)

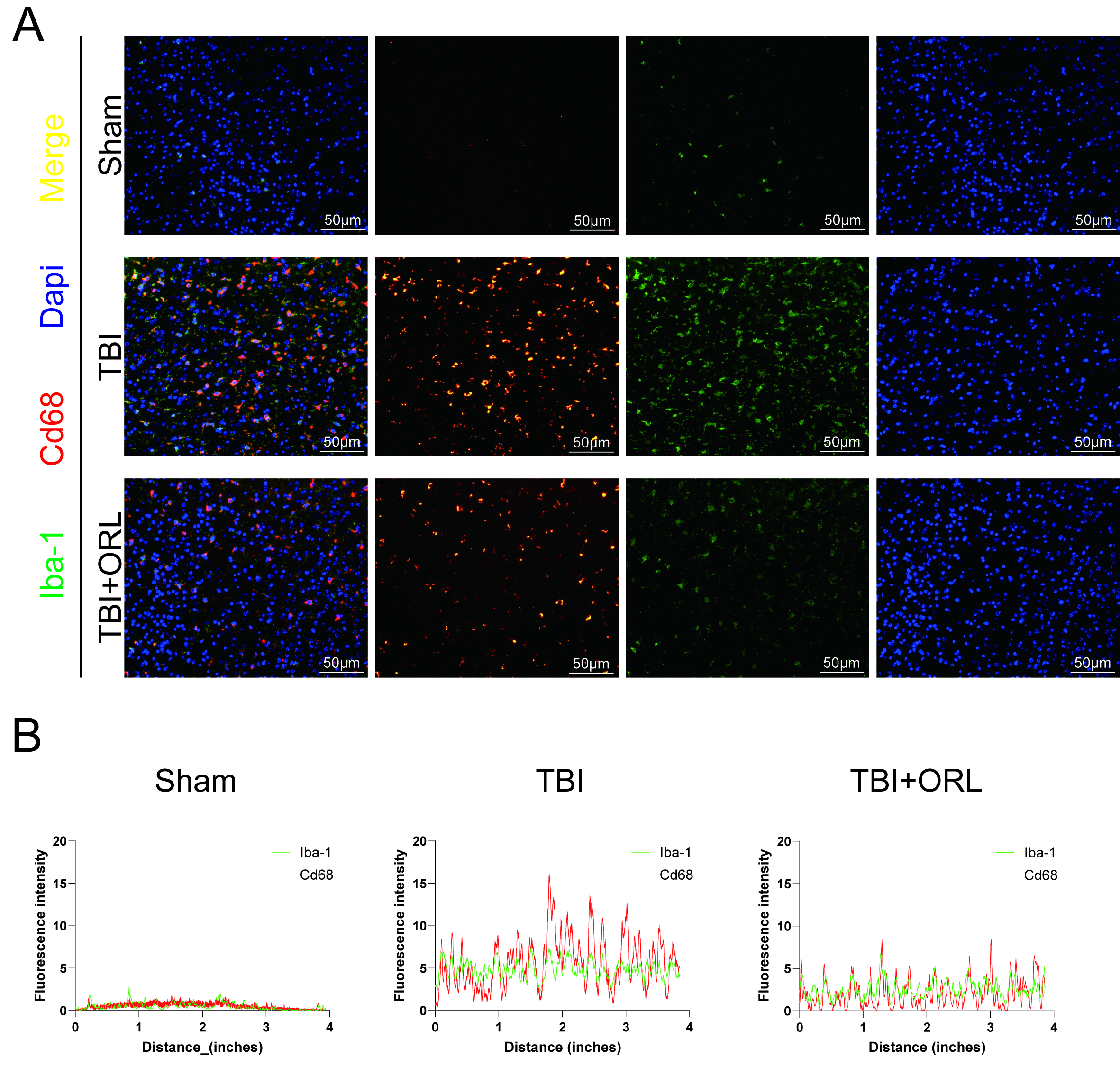

Supplement: Supplementary file 1 [file cells-14-01469-s001.zip › Fig S4.tif]

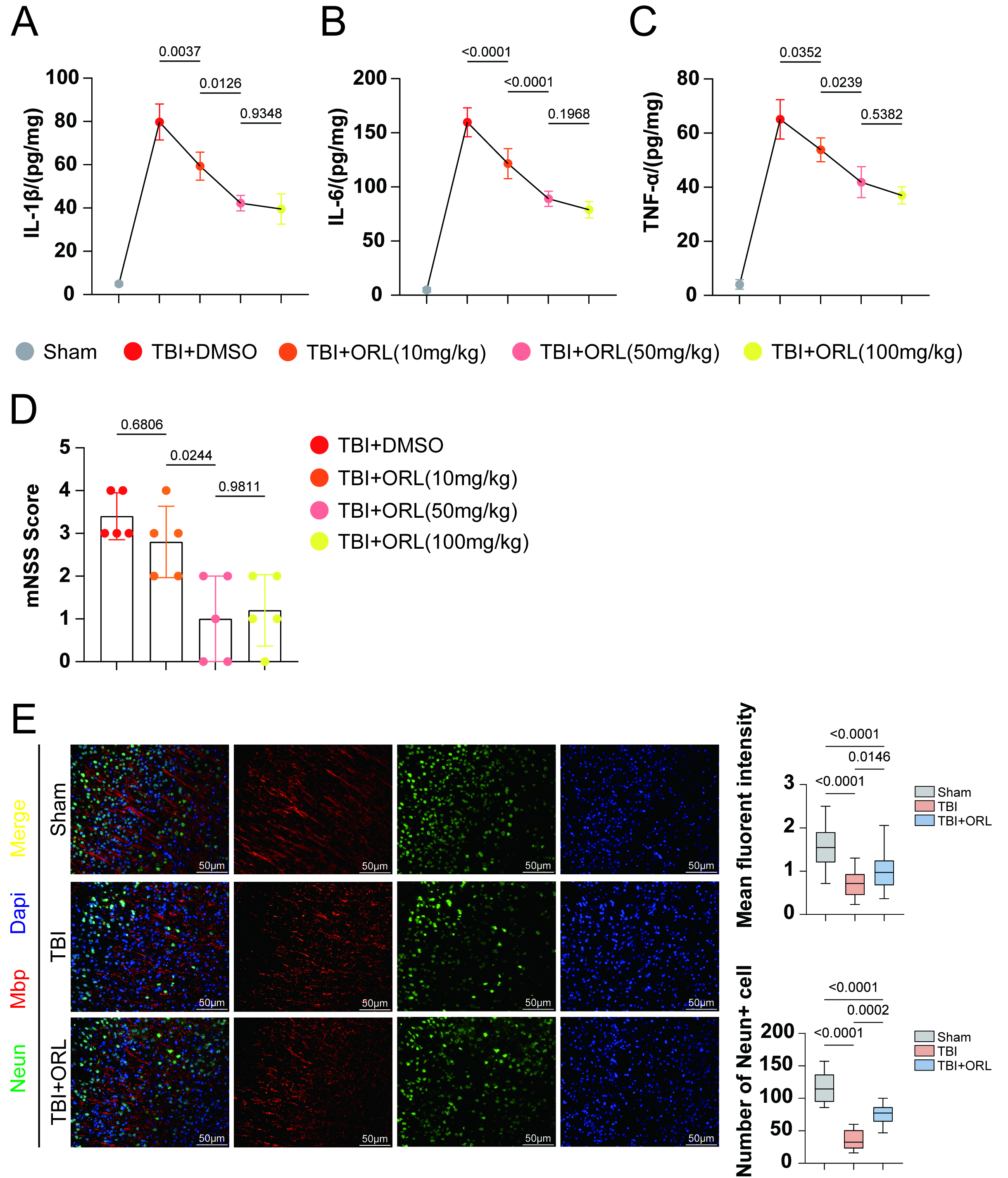

Supplement: Supplementary file 1 [file cells-14-01469-s001.zip › Fig S1.tif]

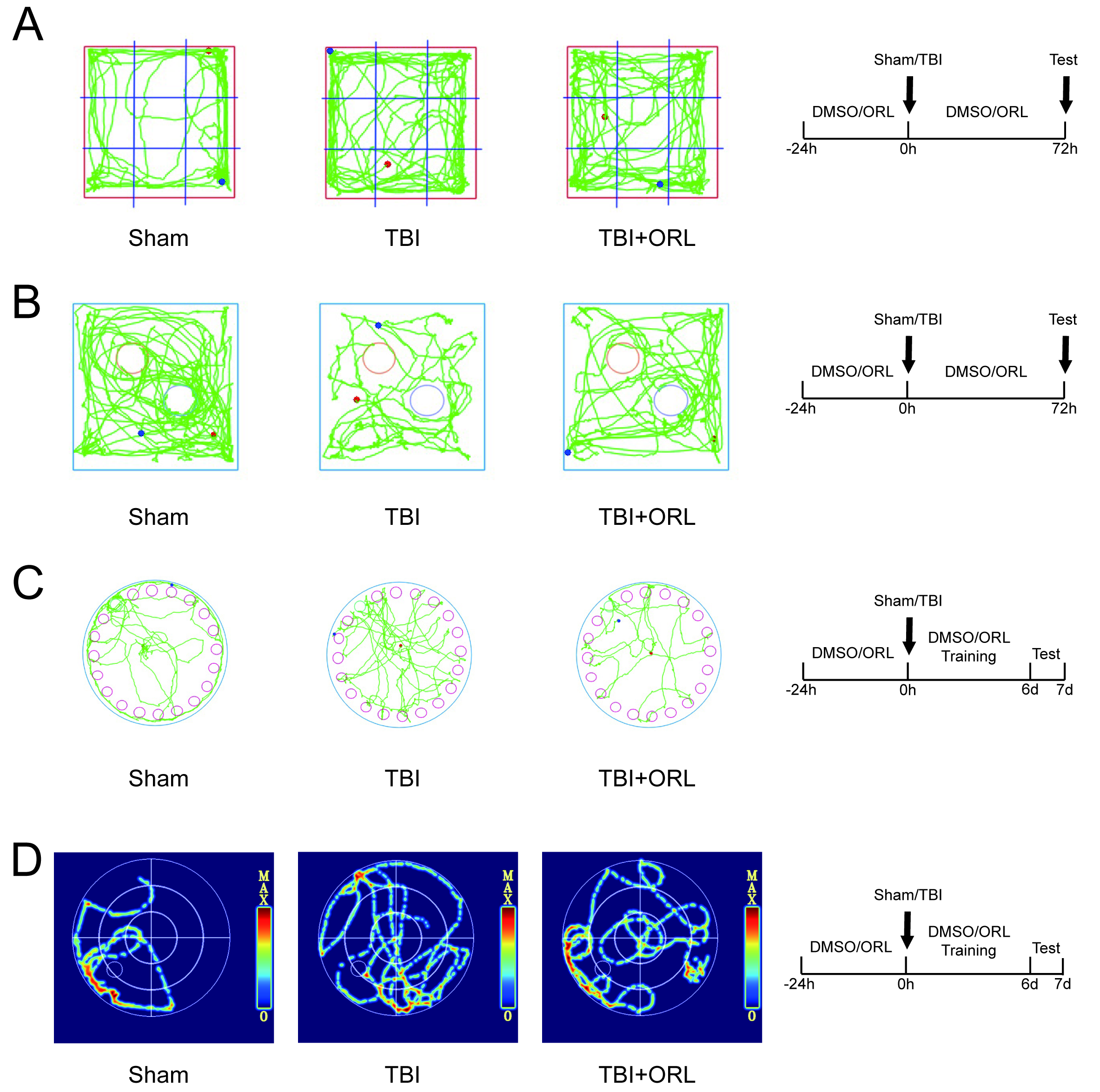

Supplement: Supplementary file 1 [file cells-14-01469-s001.zip › Fig S2.tif]

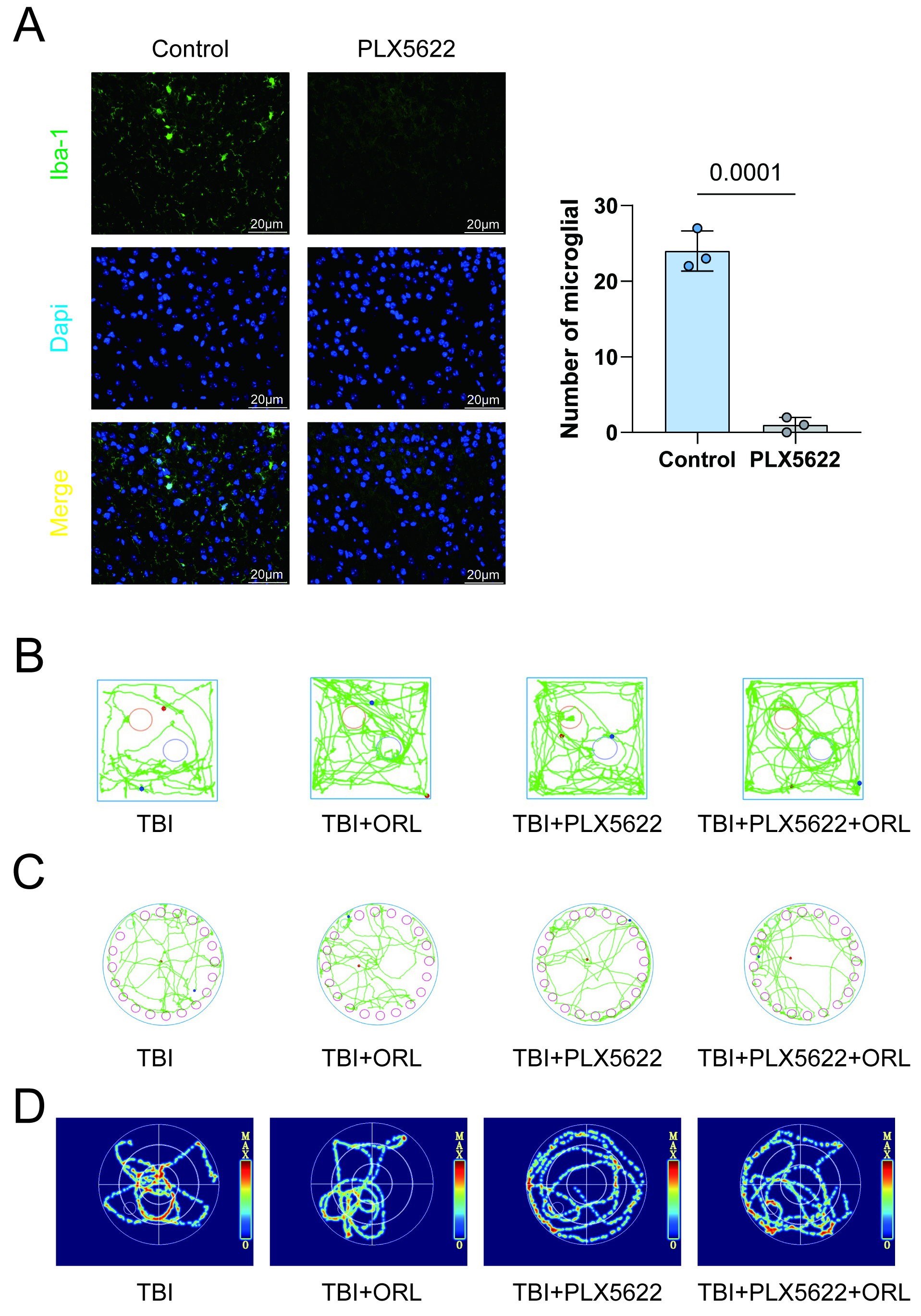

Supplement: Supplementary file 1 [file cells-14-01469-s001.zip › Fig S3.tif]
